# Supplementary material for: Biological Strategies of Invasive Bark Beetles and Borers Species
Source: Insects. 2021 Apr 20;12(4):367. doi: 10.3390/insects12040367 (PMC8074309; doi:10.3390/insects12040367)
Supplement: Supplementary file 1 [file insects-12-00367-s001.zip › Figure S1.pdf]

Layout of species in the dimensions 1 and 2 of the factor space (FAMD of invasive species)

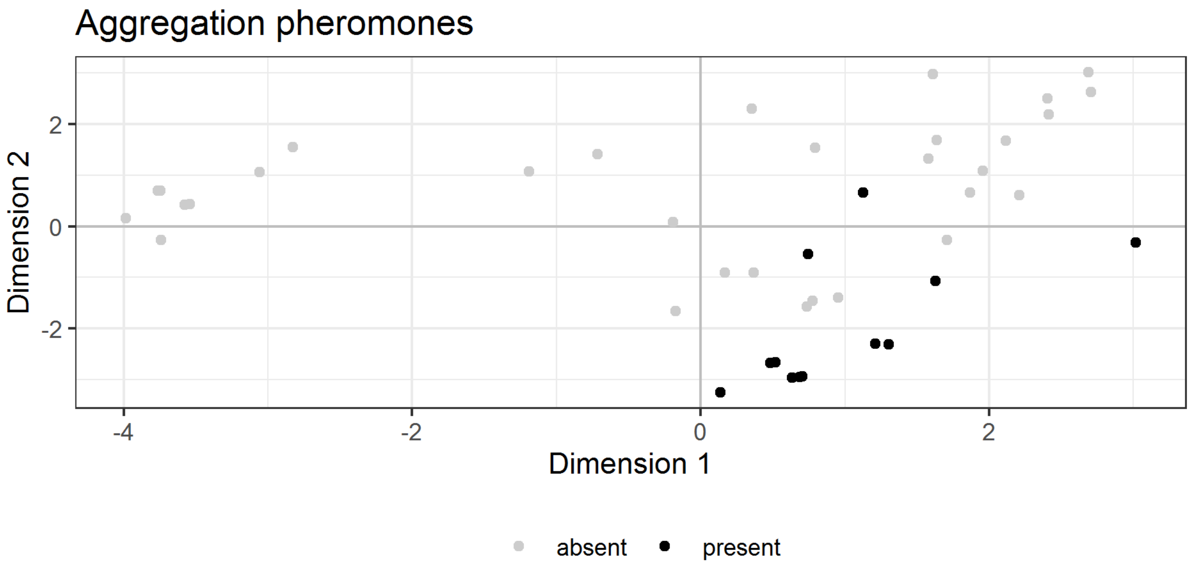

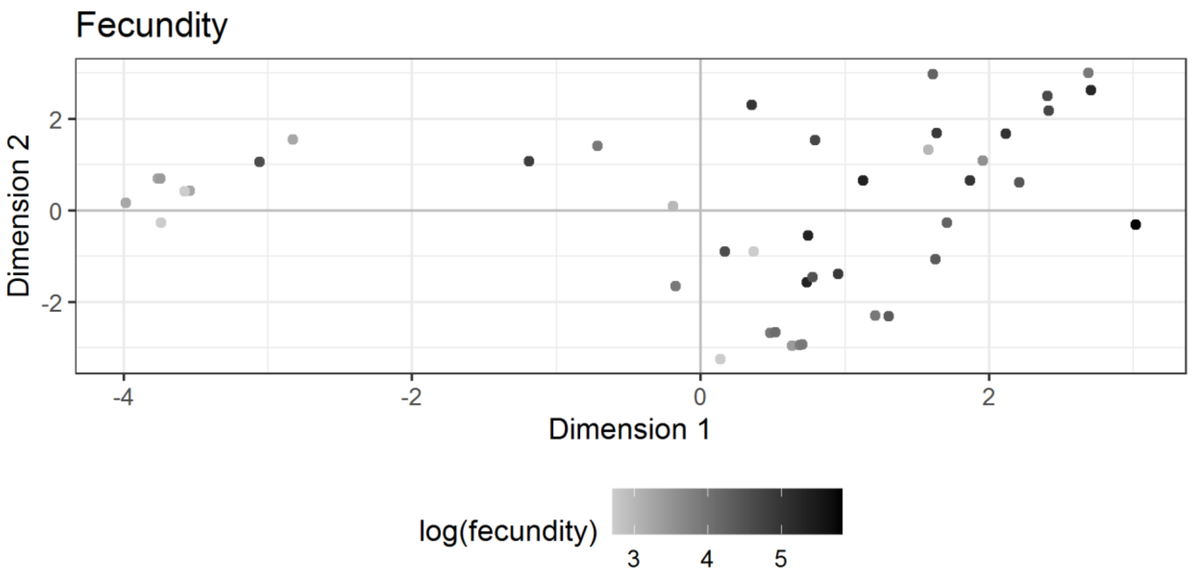

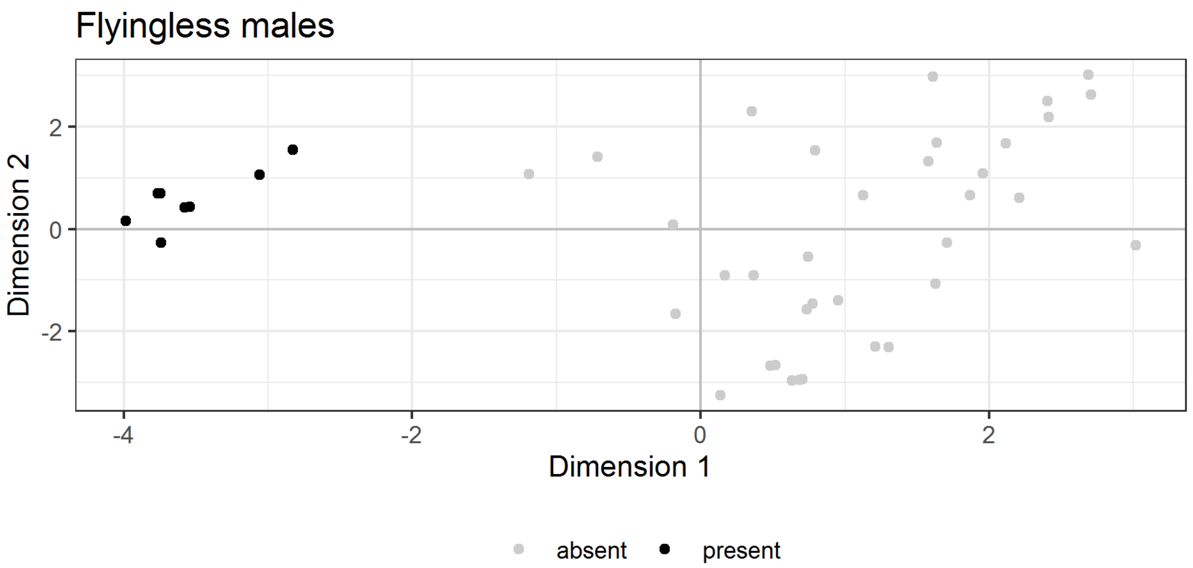

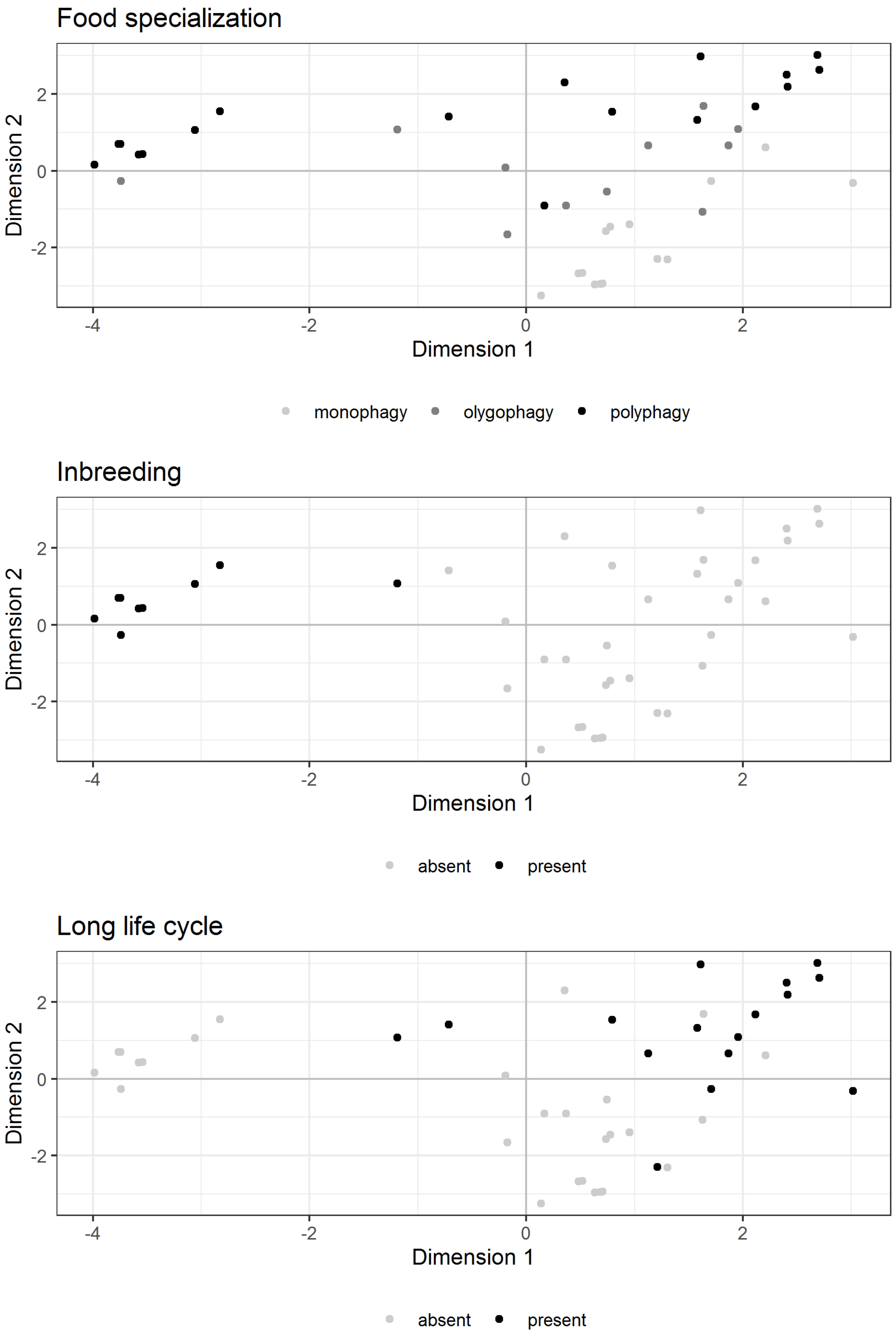
Food specialization
Dimension 2
Dimension 1
monophagy
olygophagy
polyphagy
Inbreeding
Dimension 2
Dimension 1
absent
present
Long life cycle
Dimension 2
Dimension 1
absent
present

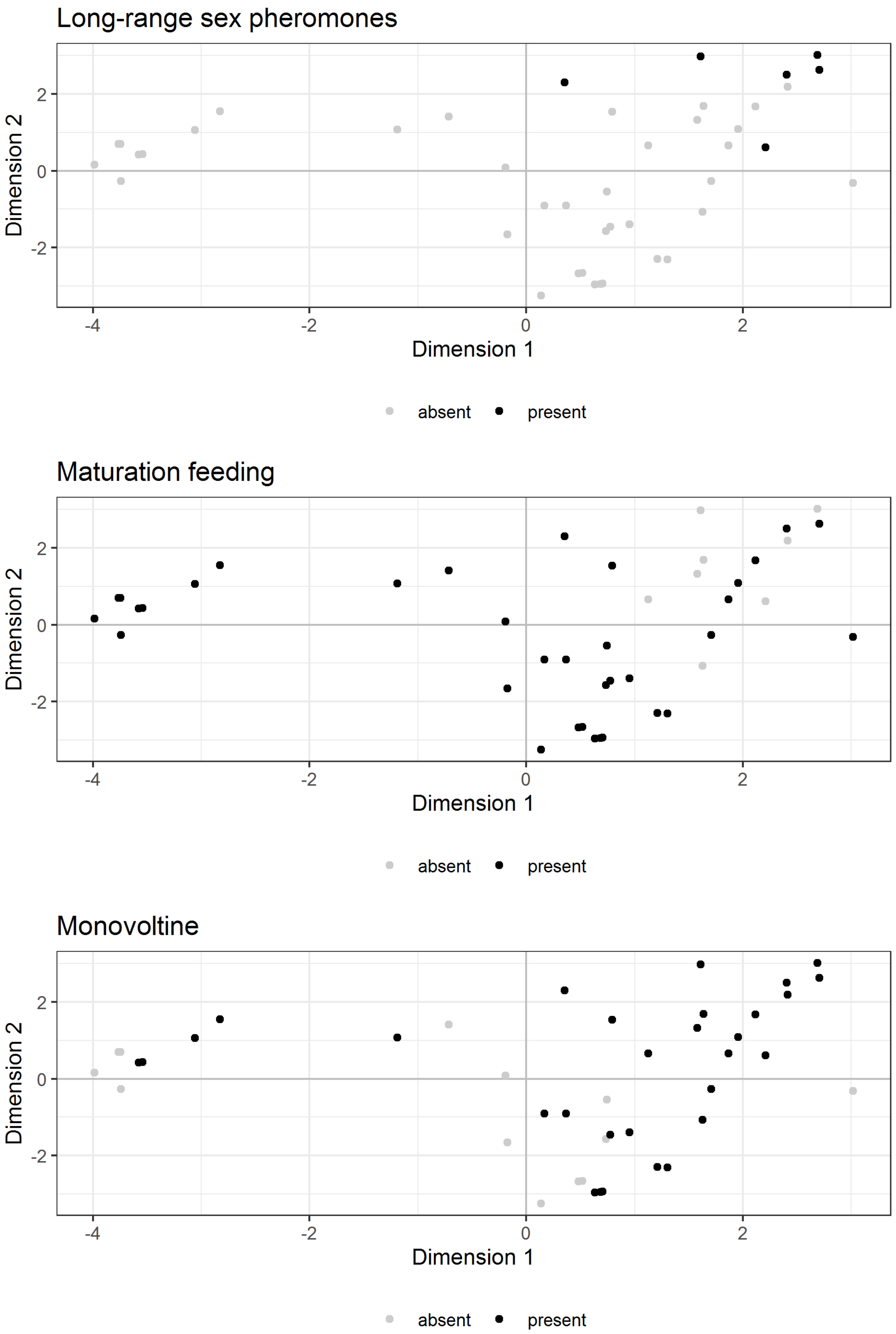
Long-range sex pheromones
Dimension 1
Dimension 2
absent
present
Maturation feeding
Dimension 1
Dimension 2
absent
present
Monovoltine
Dimension 1
Dimension 2
absent
present

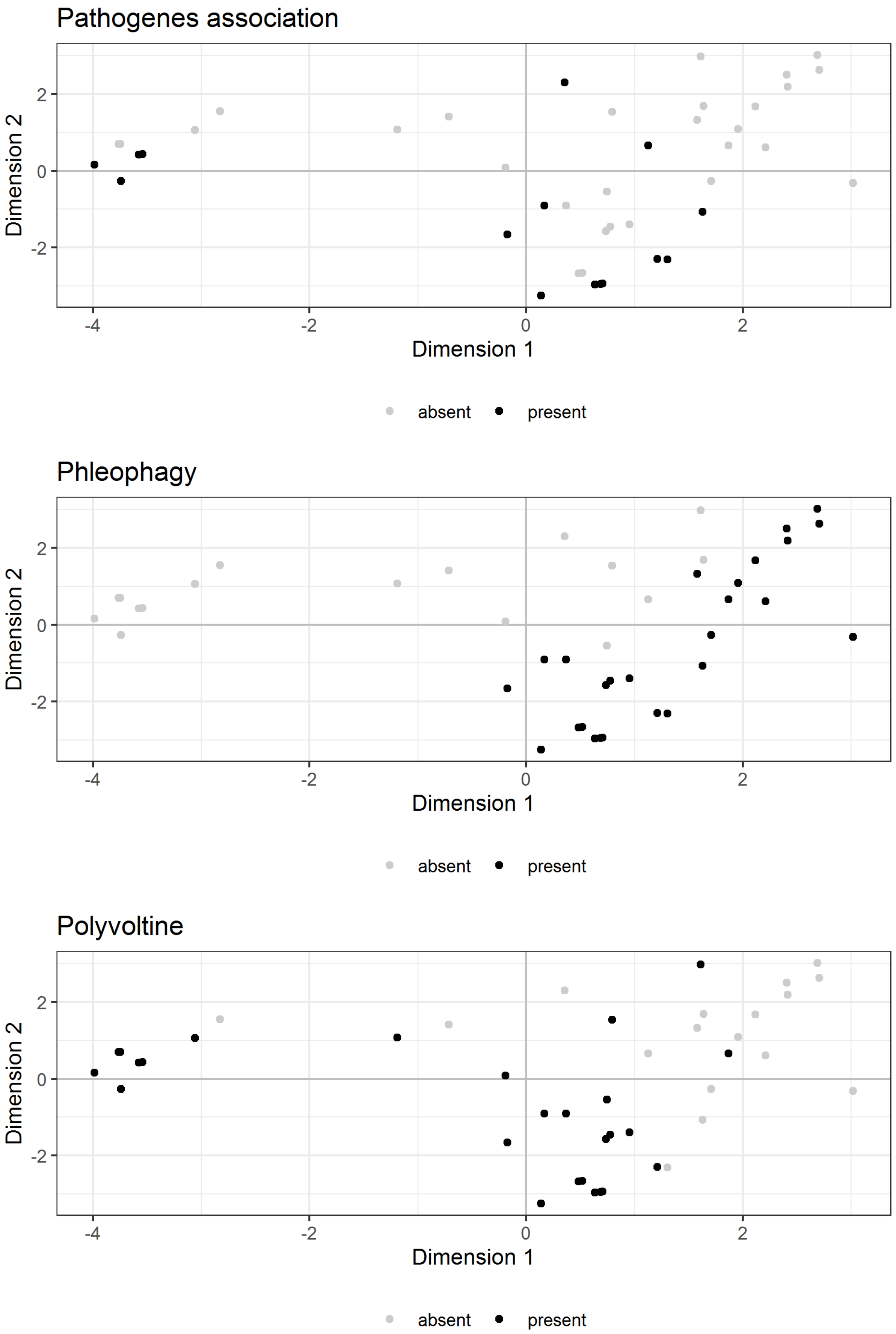
Pathogenes association
Dimension 2
Dimension 1
absent
present
Phleophagy
Dimension 2
Dimension 1
absent
present
Polyvoltine
Dimension 2
Dimension 1
absent
present

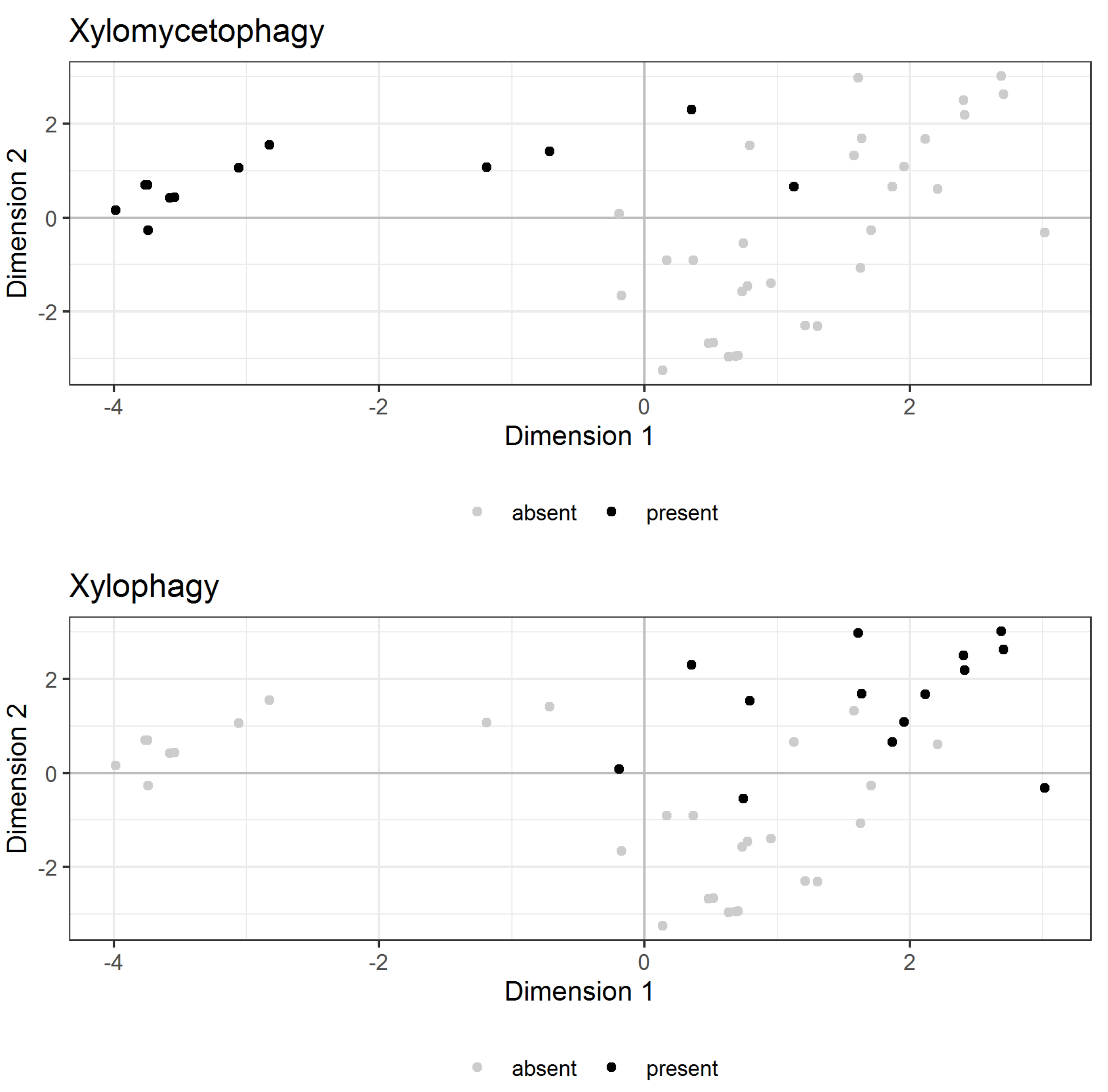

Xylomycetophagy
Dimension 2
Dimension 1
absent
present
Xylophagy
Dimension 2
Dimension 1
absent
present

Layout of species in the dimensions 1 and 3 of the factor space (FAMD of invasive species)

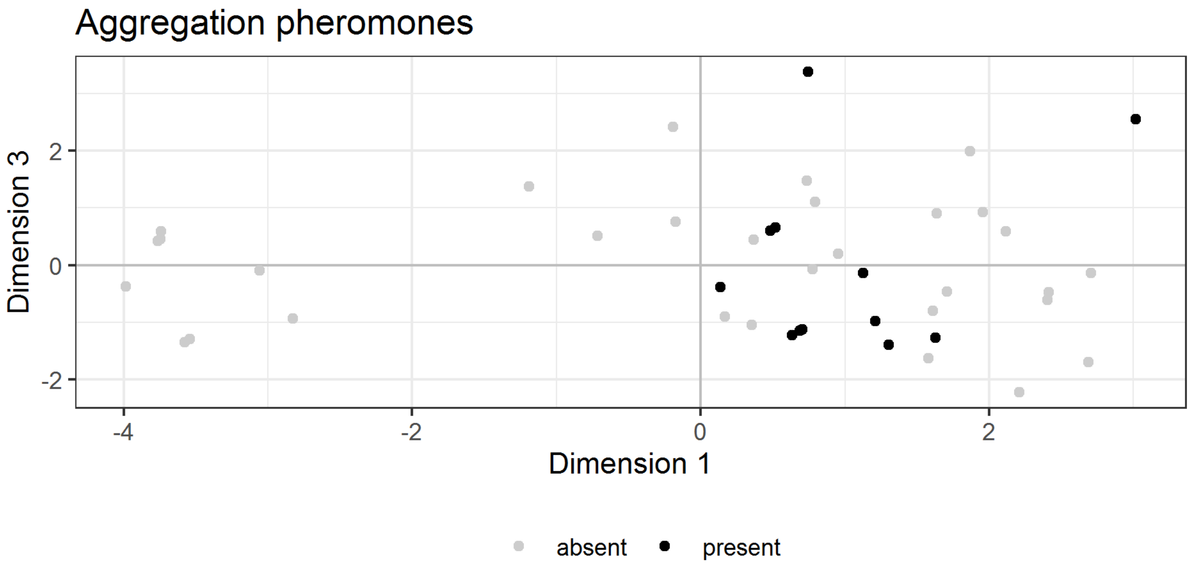

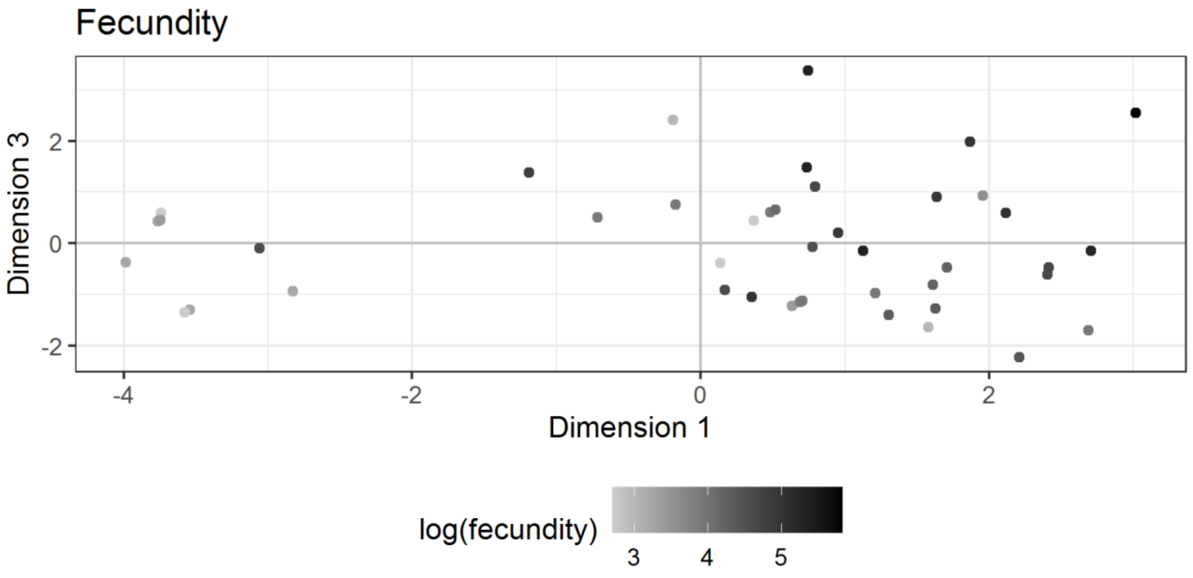

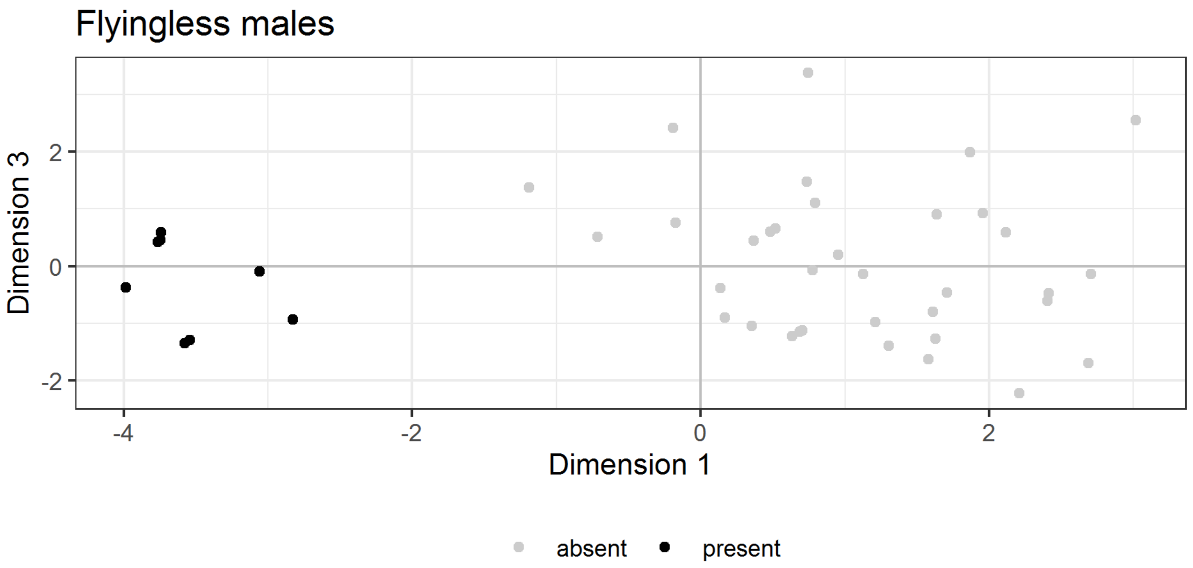

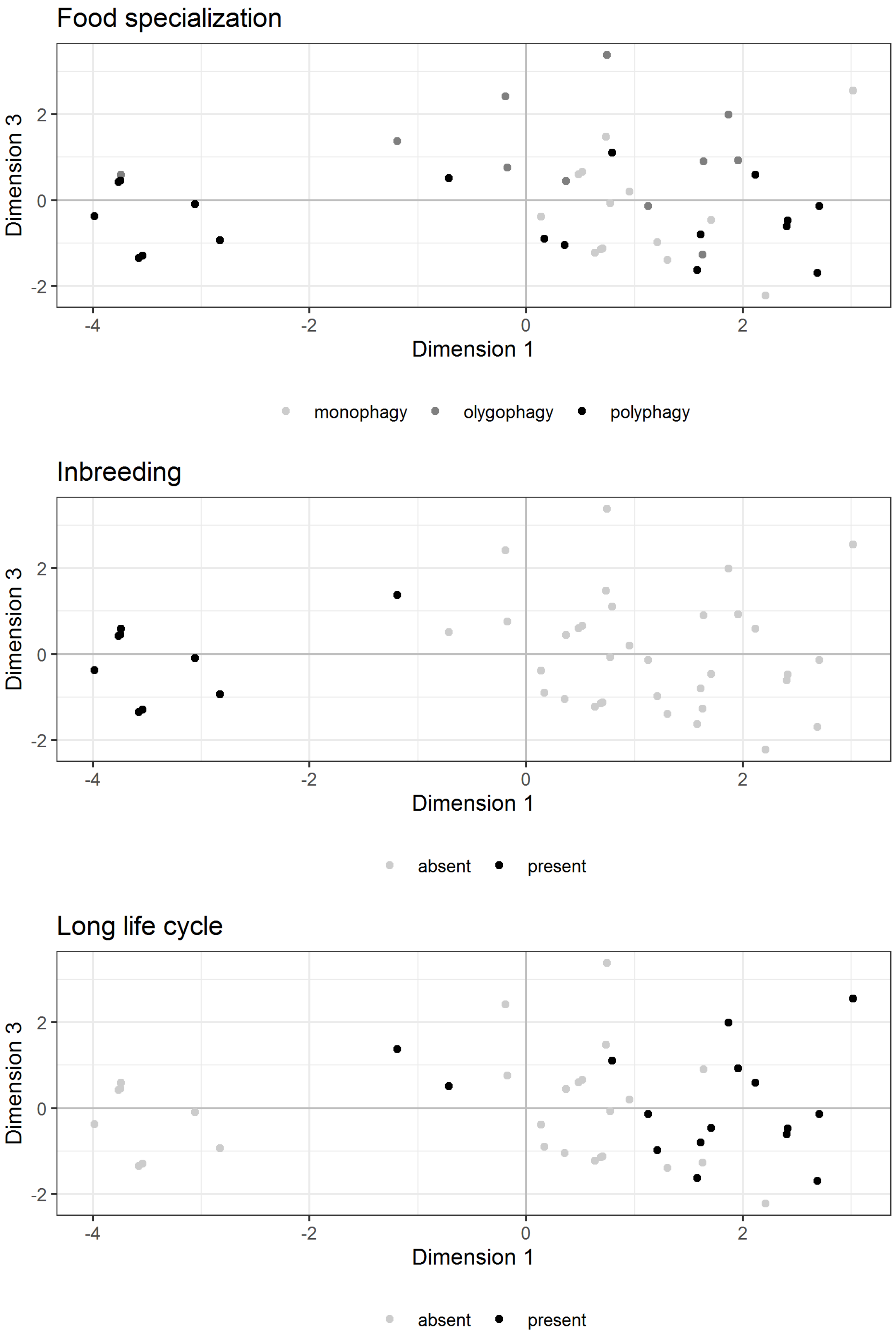
Food specialization
Dimension 3
Dimension 1
monophagy
olygophagy
polyphagy
Inbreeding
Dimension 3
Dimension 1
absent
present
Long life cycle
Dimension 3
Dimension 1
absent
present

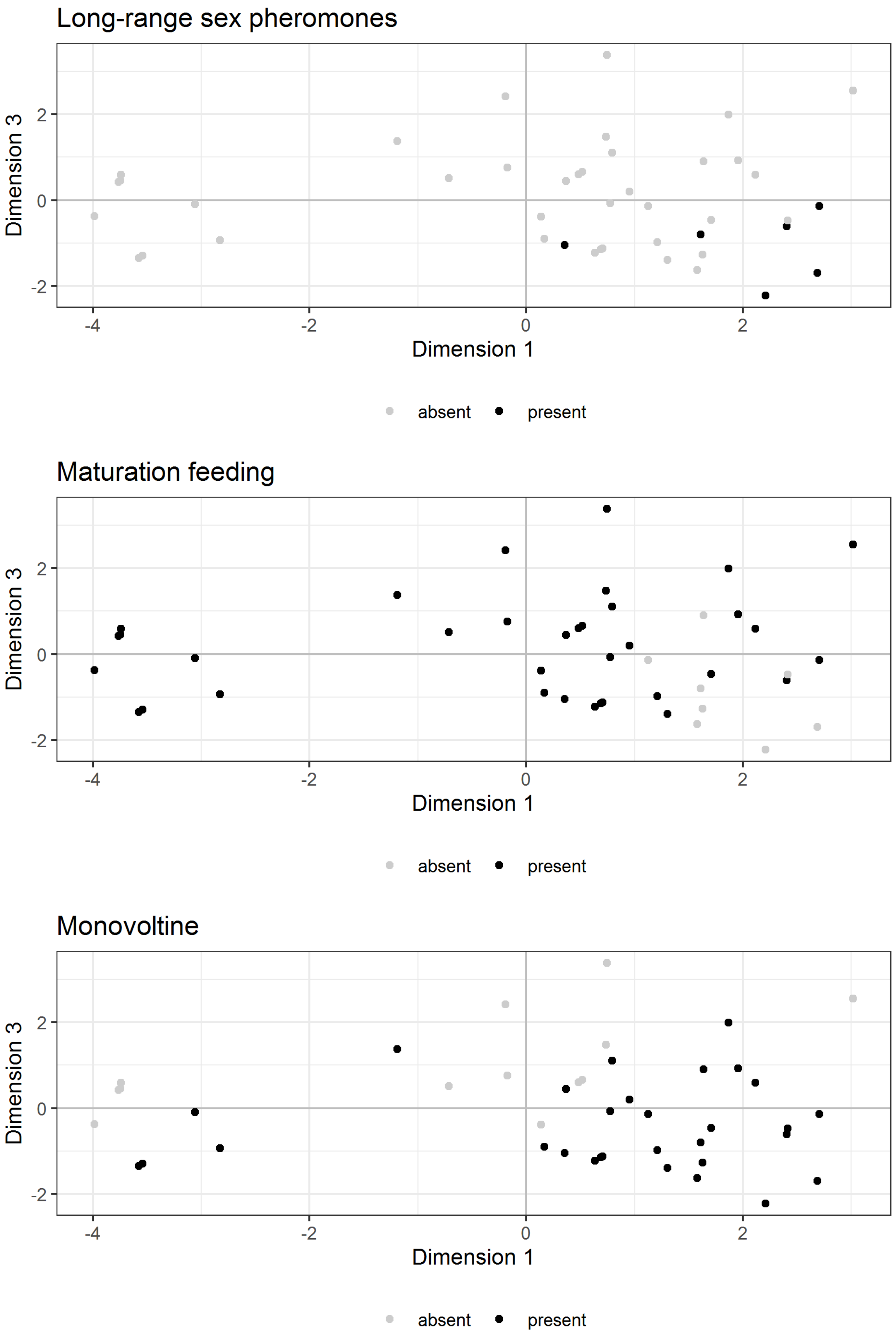
Long-range sex pheromones
Dimension 3
Dimension 1
absent
present
Maturation feeding
Dimension 3
Dimension 1
absent
present
Monovoltine
Dimension 3
Dimension 1
absent
present

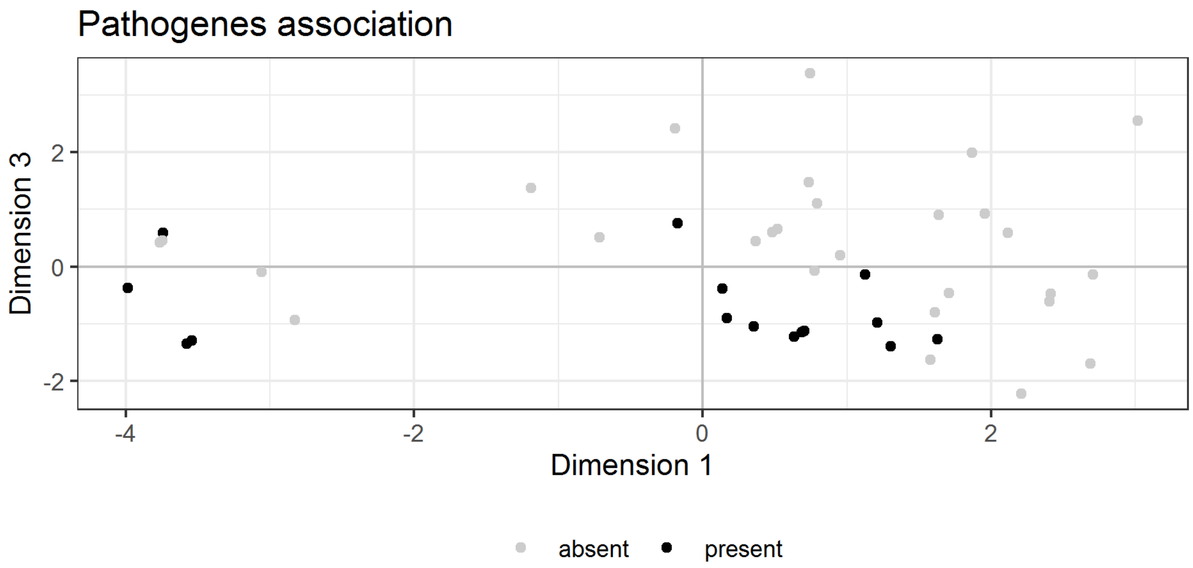
Pathogenes association
Dimension 3
Dimension 1
2
0
-2
-4
-2
0
2
absent
present

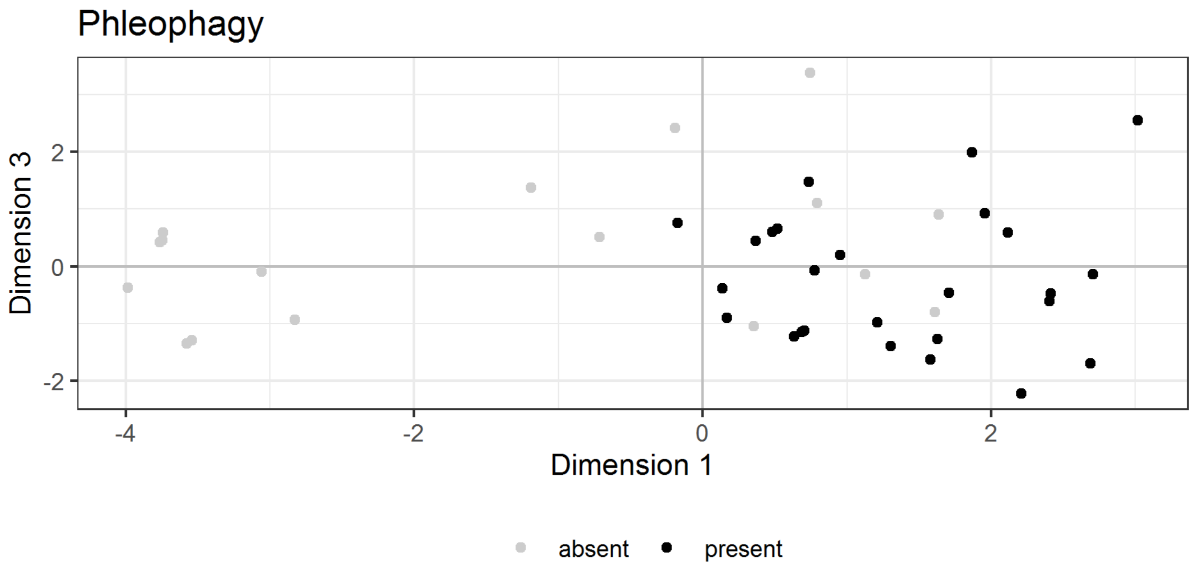
Phleophagy
Dimension 3
Dimension 1
2
0
-2
-4
-2
0
2
absent
present

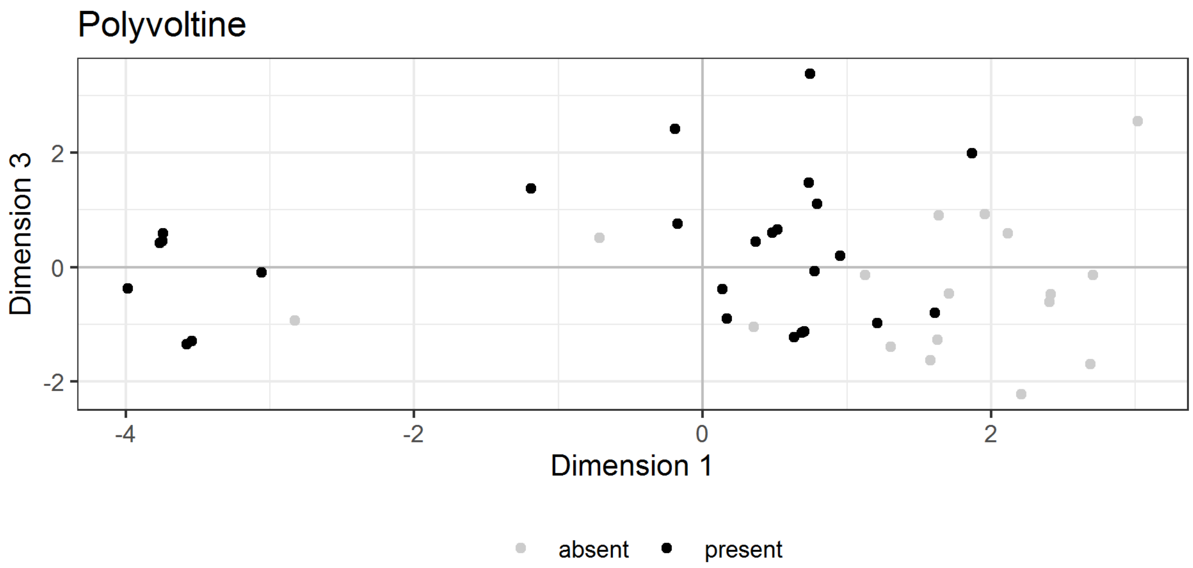
Polyvoltine
Dimension 3
Dimension 1
2
0
-2
-4
-2
0
2
absent
present

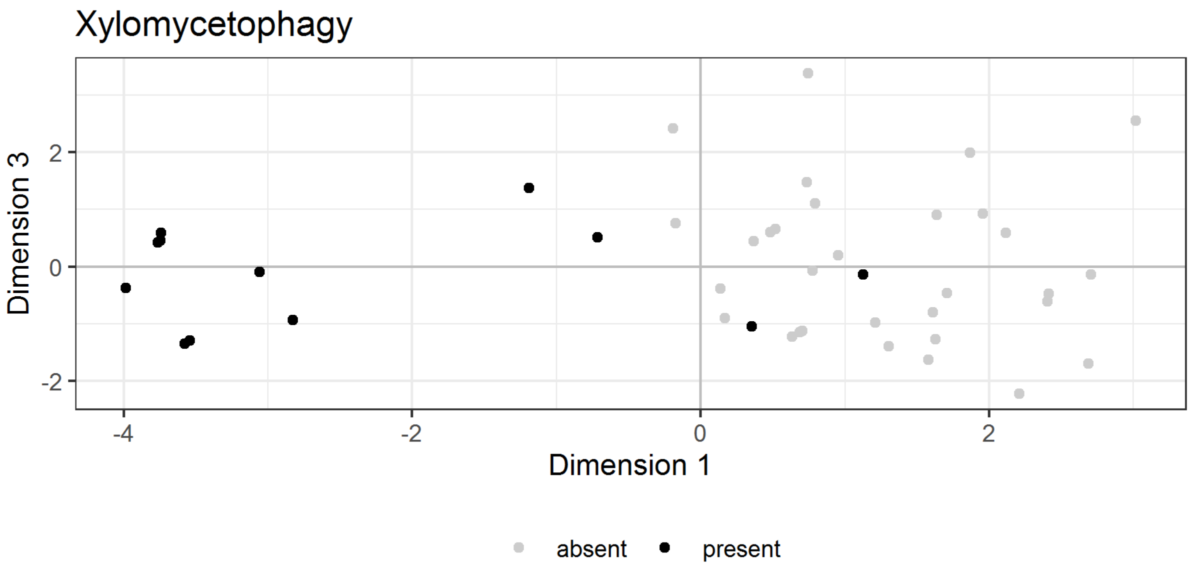
Xylomycetophagy
Dimension 3
2
0
-2
-4
-2
0
2
Dimension 1
absent
present

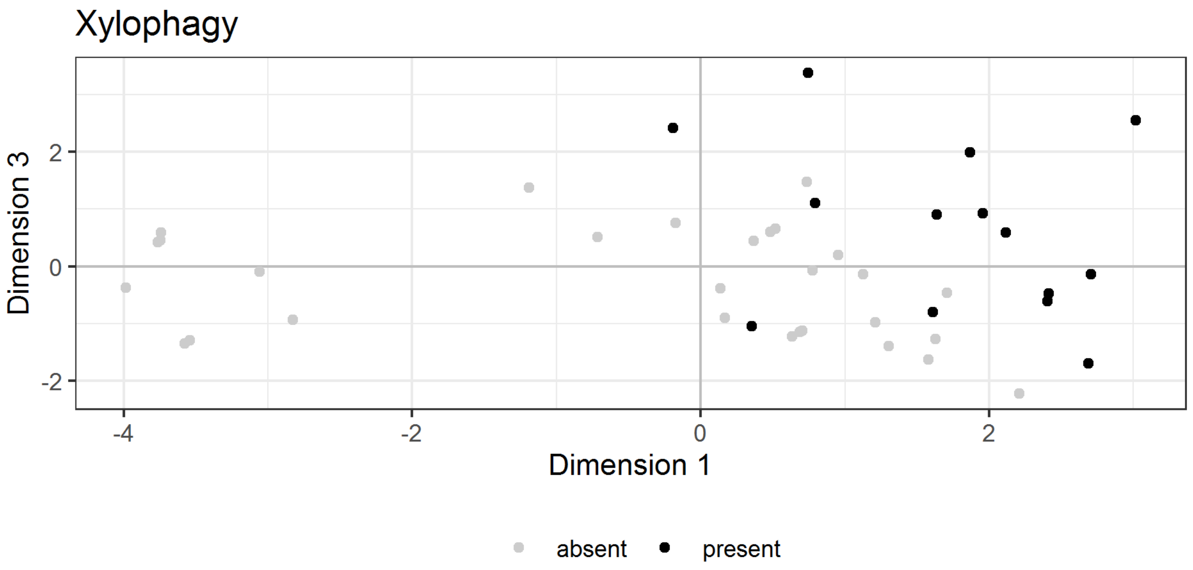
Xylophagy
Dimension 3
2
0
-2
-4
-2
0
2
Dimension 1
absent
present

Layout of species in the dimensions 3 and 4 of the factor space (FAMD of invasive vs. non-invasive species)

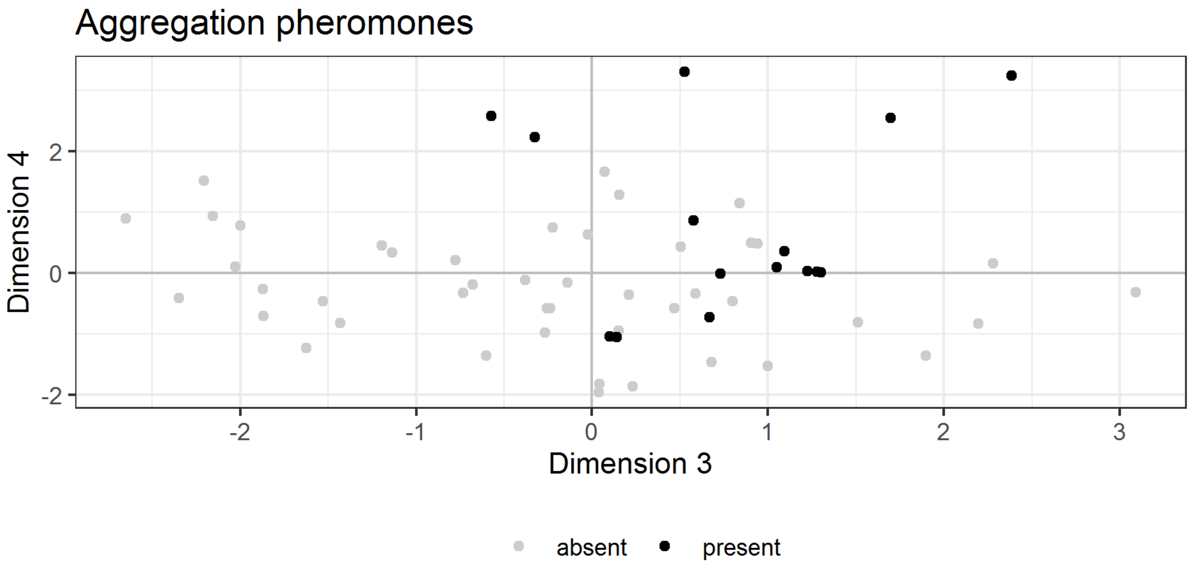

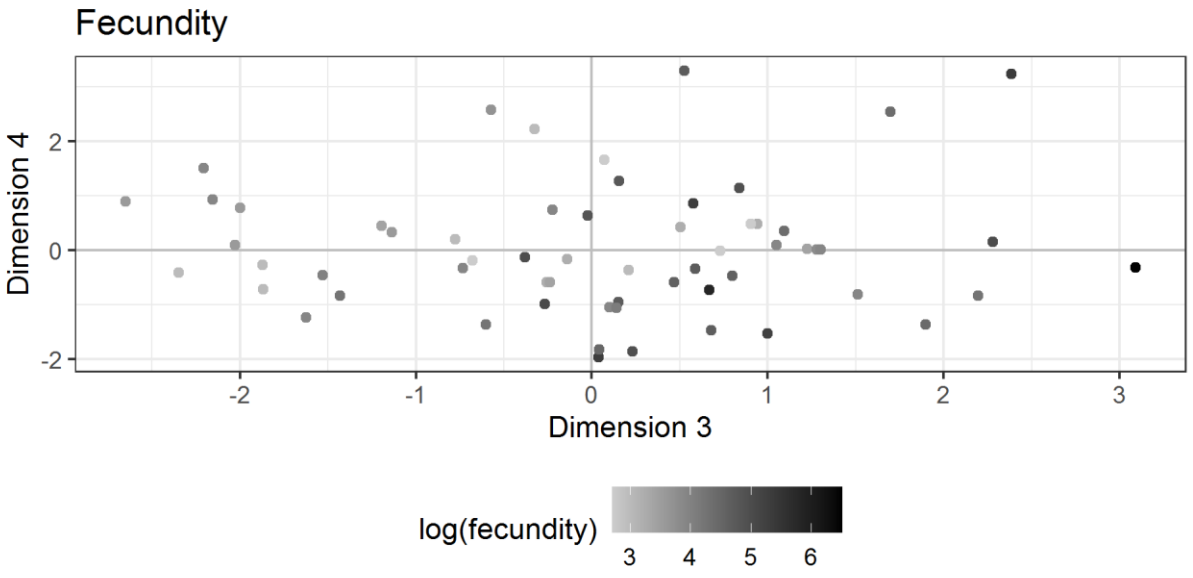

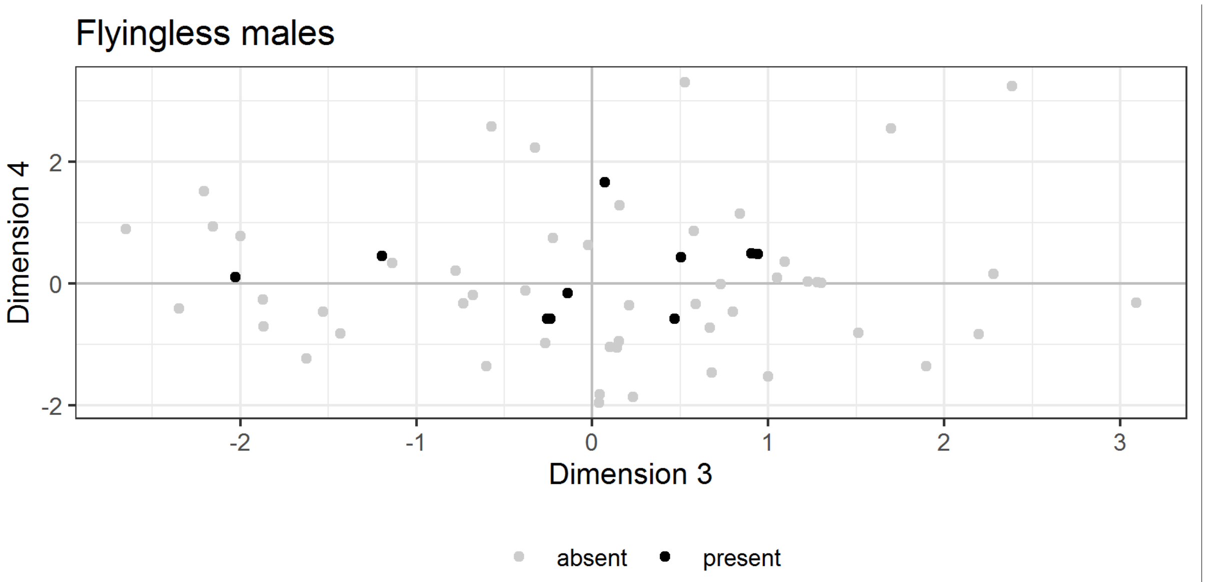

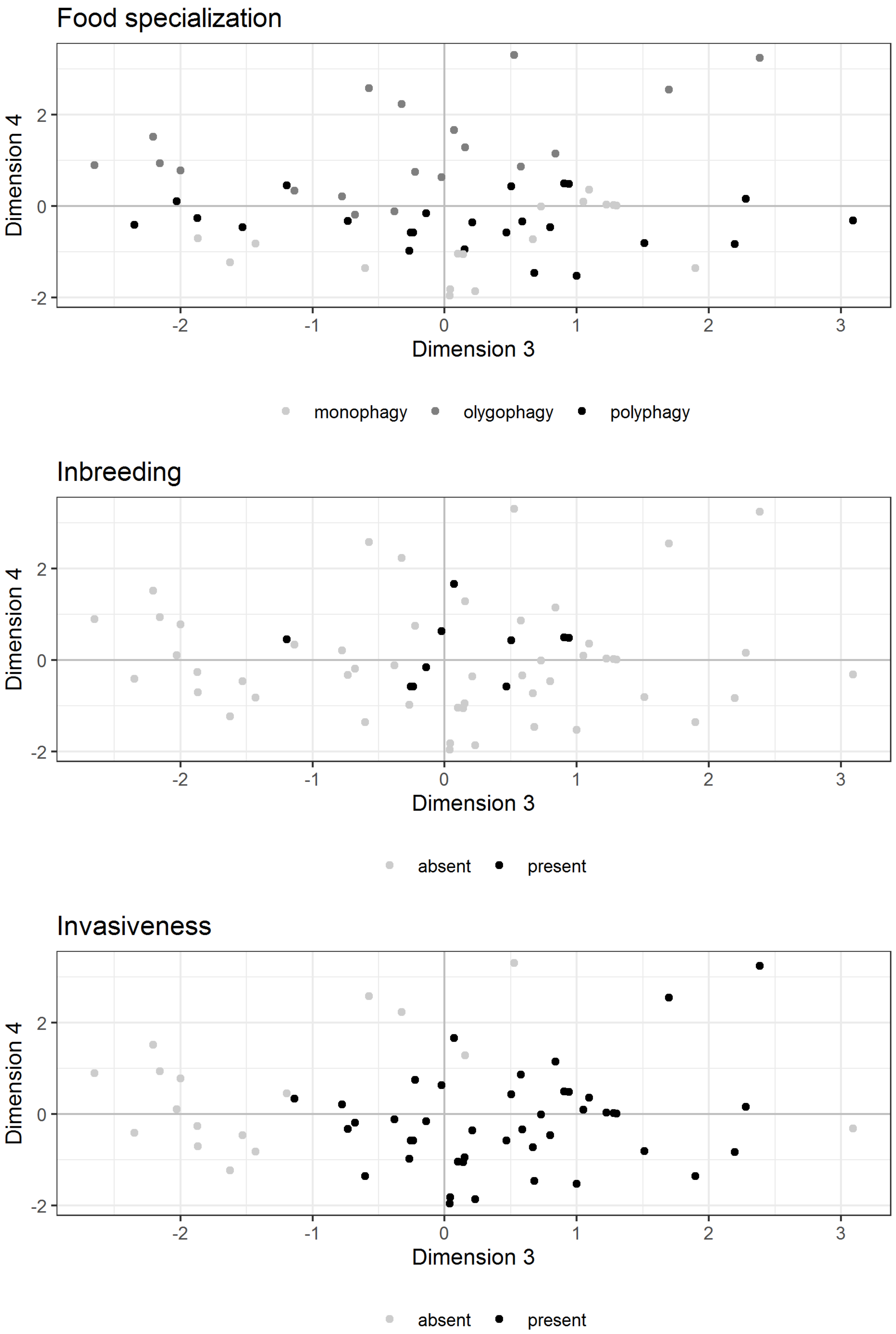
Food specialization
Dimension 4
Dimension 3
monophagy
olygophagy
polyphagy
Inbreeding
Dimension 4
Dimension 3
absent
present
Invasiveness
Dimension 4
Dimension 3
absent
present

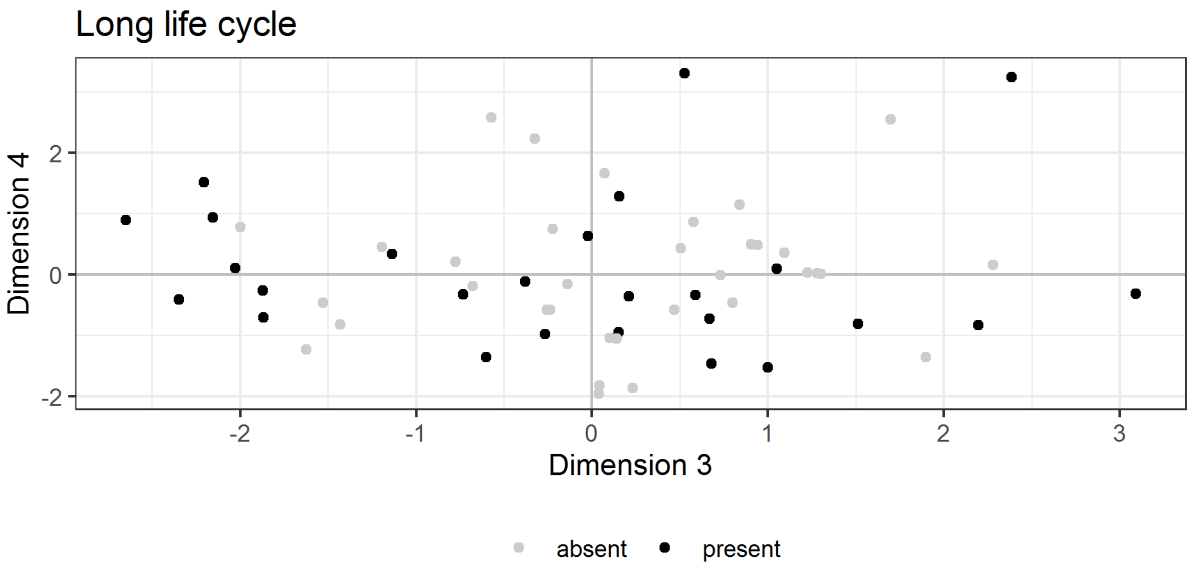

Long life cycle
Dimension 4
Dimension 3
2
0
-2
-1
1
3
absent
present

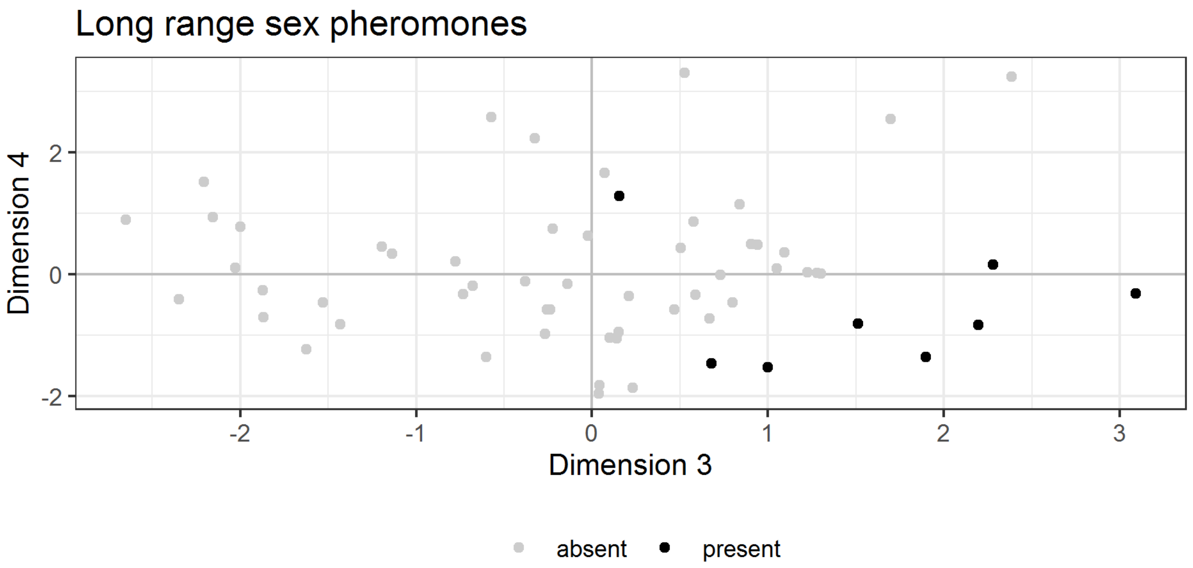

Long range sex pheromones
Dimension 4
Dimension 3
2
0
-2
-1
1
3
absent
present

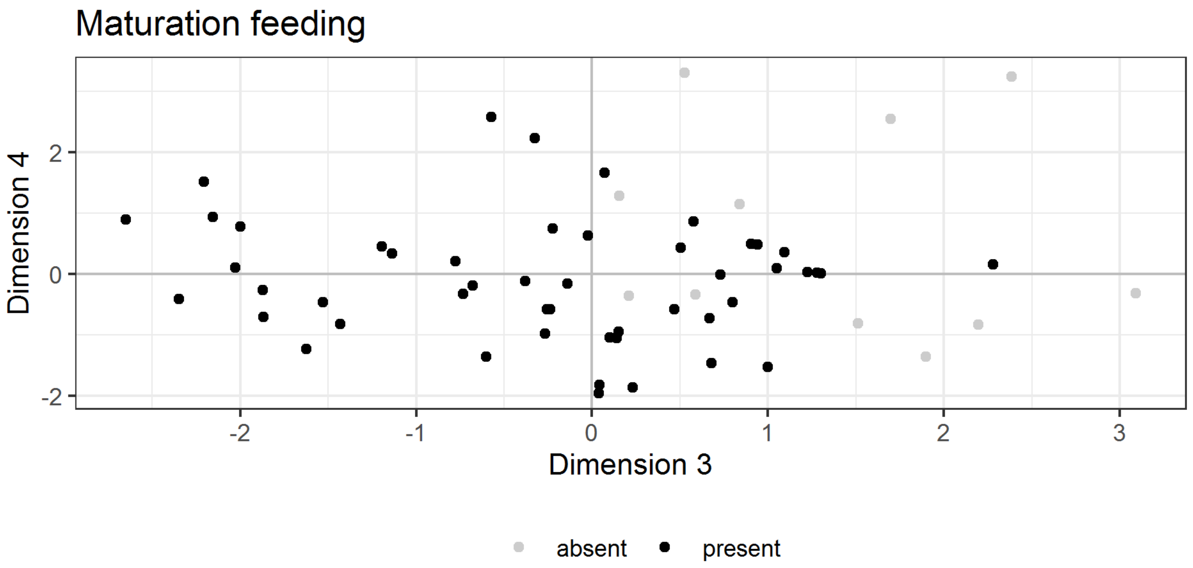

Maturation feeding
Dimension 4
Dimension 3
2
0
-2
-1
1
3
absent
present

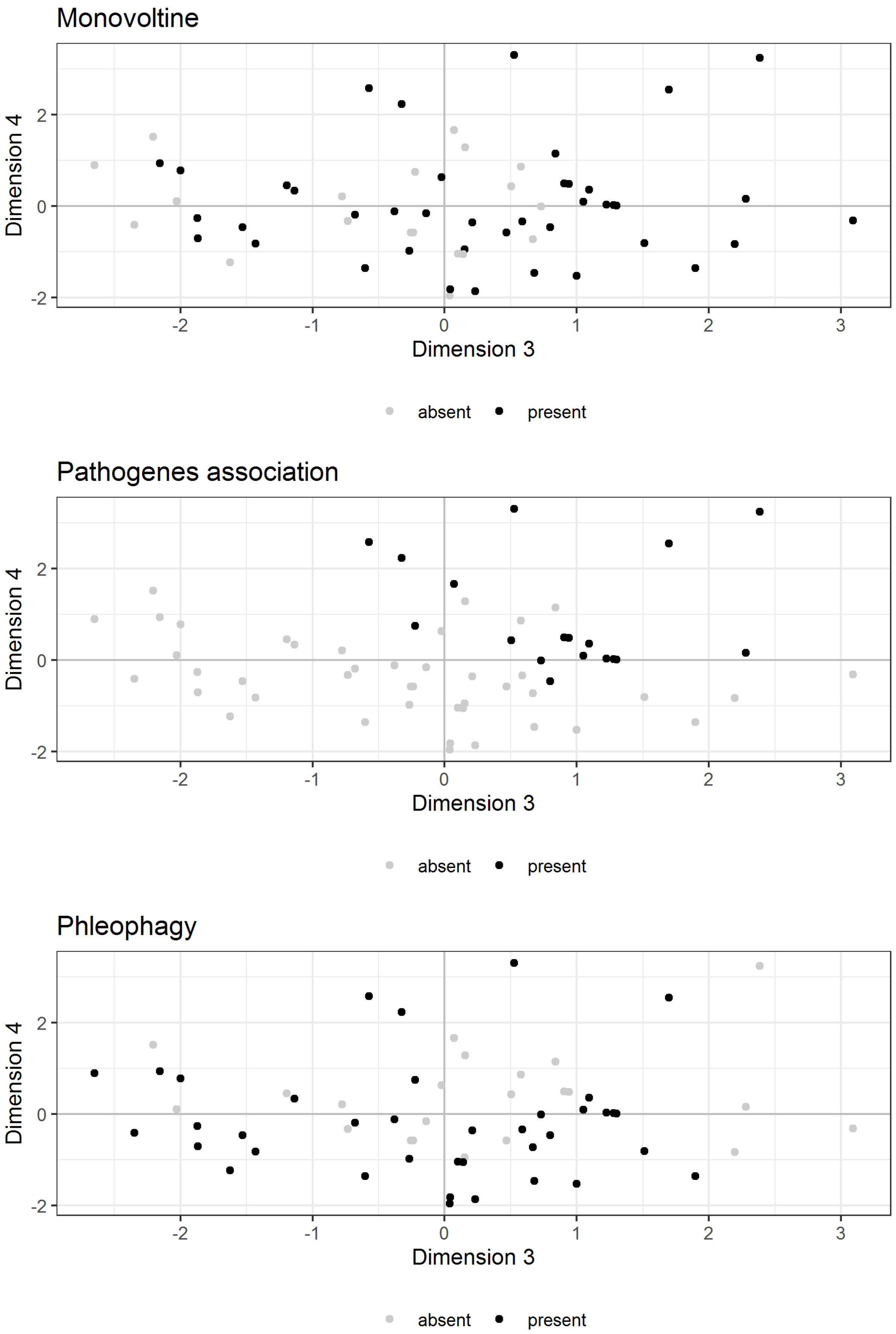
Monovoltine
Dimension 4
Dimension 3
absent
present
Pathogenes association
Dimension 4
Dimension 3
absent
present
Phleophagy
Dimension 4
Dimension 3
absent
present

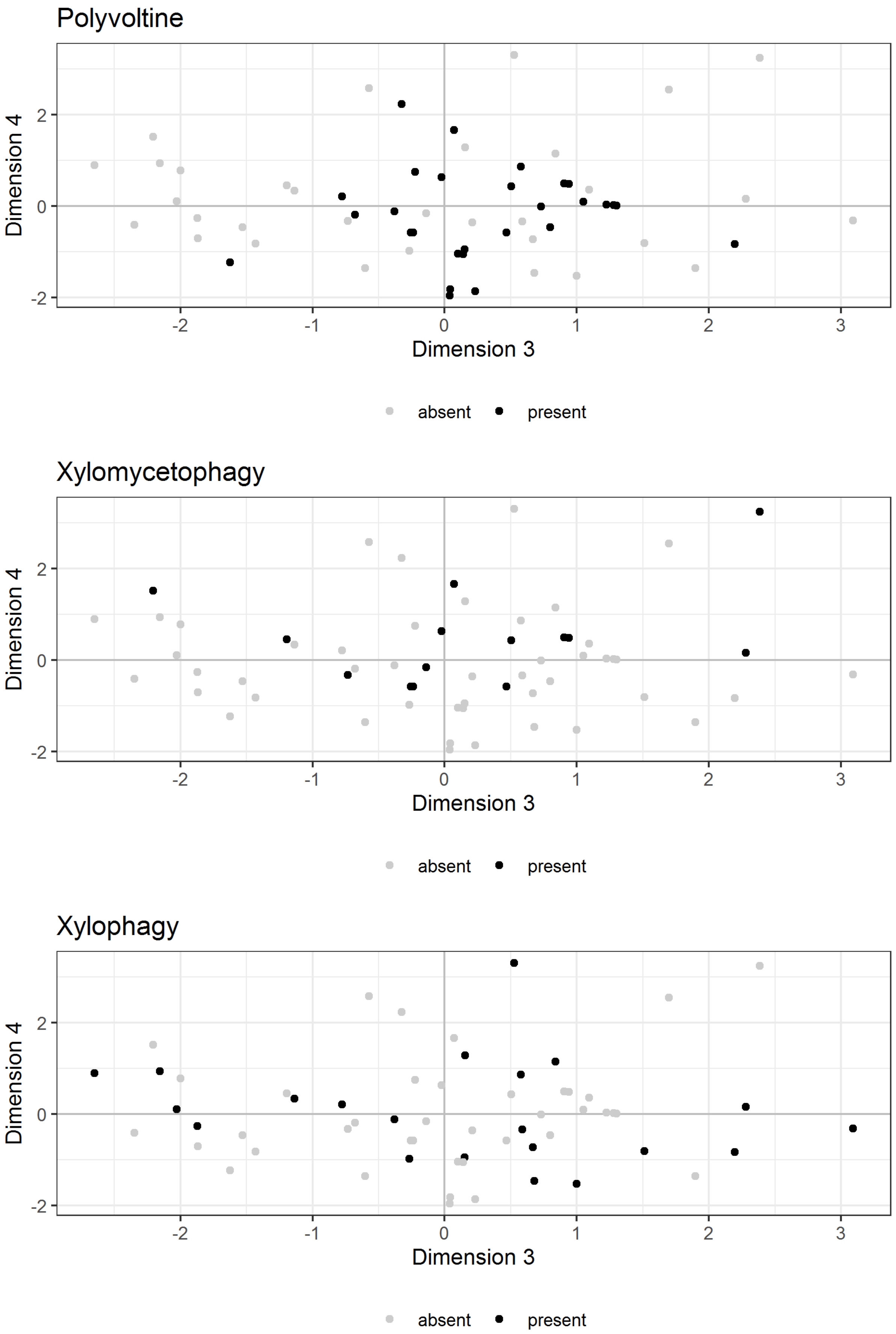
Polyvoltine
Dimension 4
Dimension 3
absent
present
Xylomycetophagy
Dimension 4
Dimension 3
absent
present
Xylophagy
Dimension 4
Dimension 3
absent
present

Layout of species in the dimensions 3 and 6 of the factor space (FAMD of invasive vs. non-invasive species)

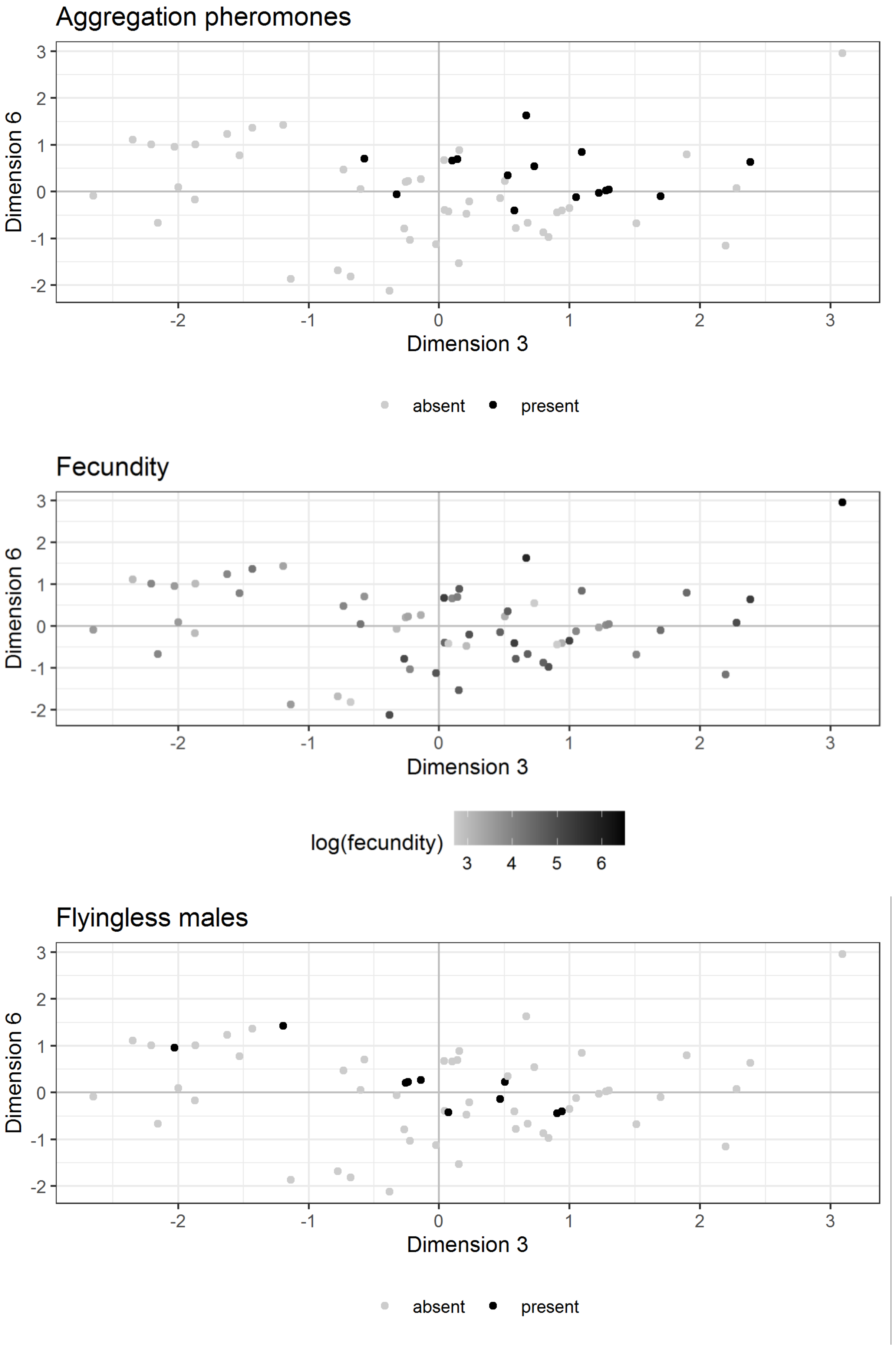

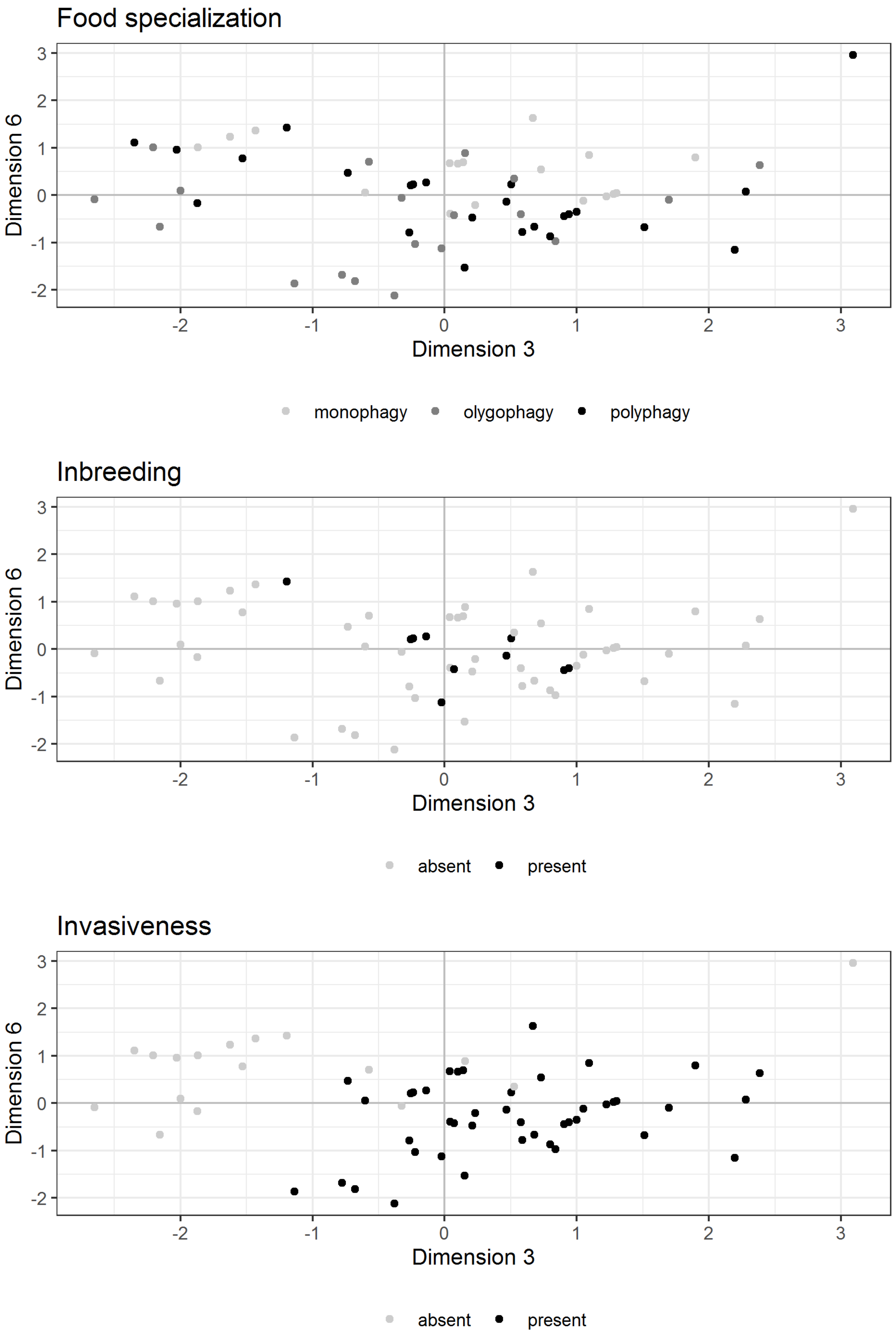
Food specialization
Dimension 6
Dimension 3
monophagy
olygophagy
polyphagy
Inbreeding
Dimension 6
Dimension 3
absent
present
Invasiveness
Dimension 6
Dimension 3
absent
present

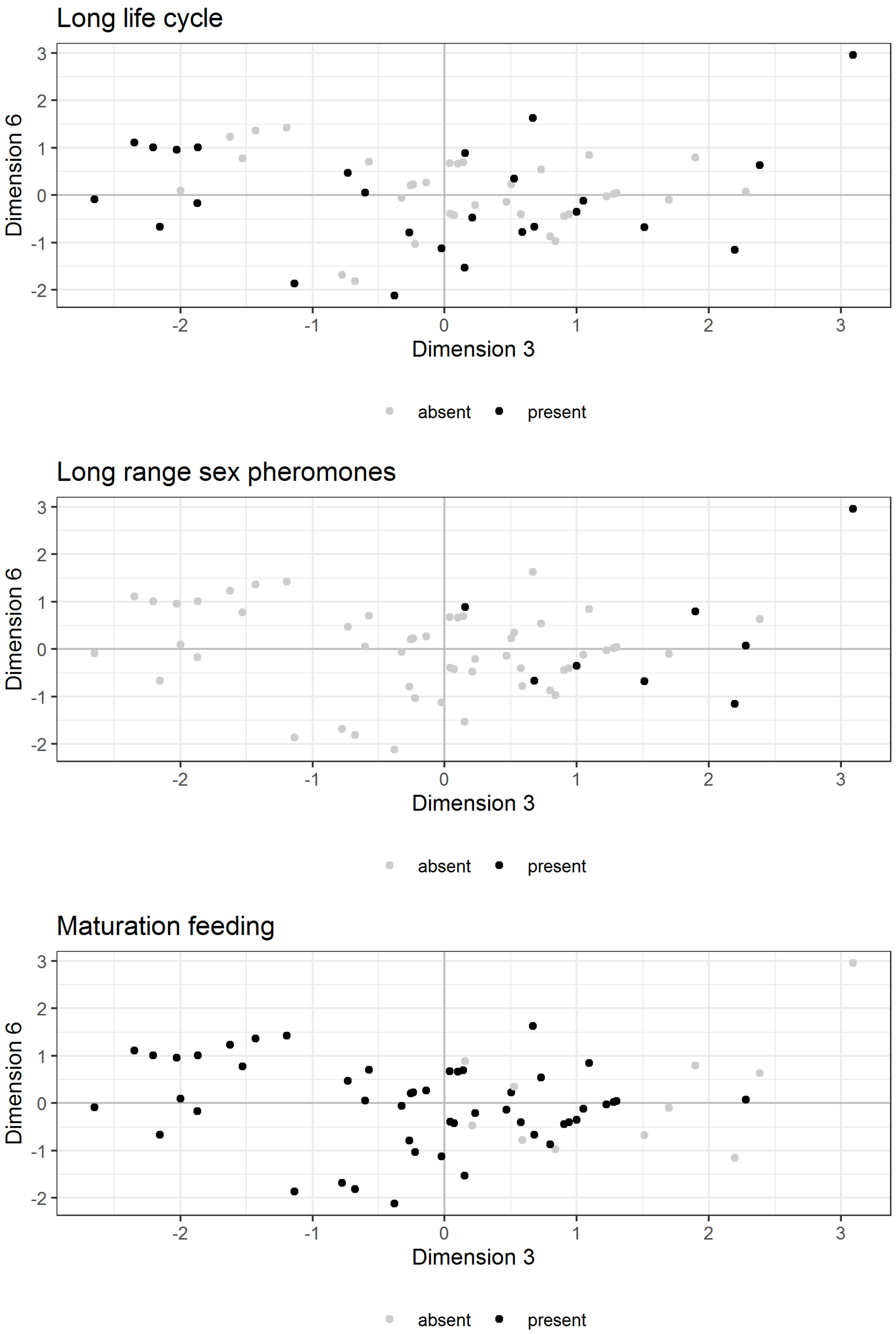
Long life cycle
Dimension 6
3
2
1
0
-1
-2
-2
-1
0
1
2
3
Dimension 3
absent
present
Long range sex pheromones
Dimension 6
Dimension 3
absent
present
Maturation feeding
Dimension 6
Dimension 3
absent
present

Monovoltine

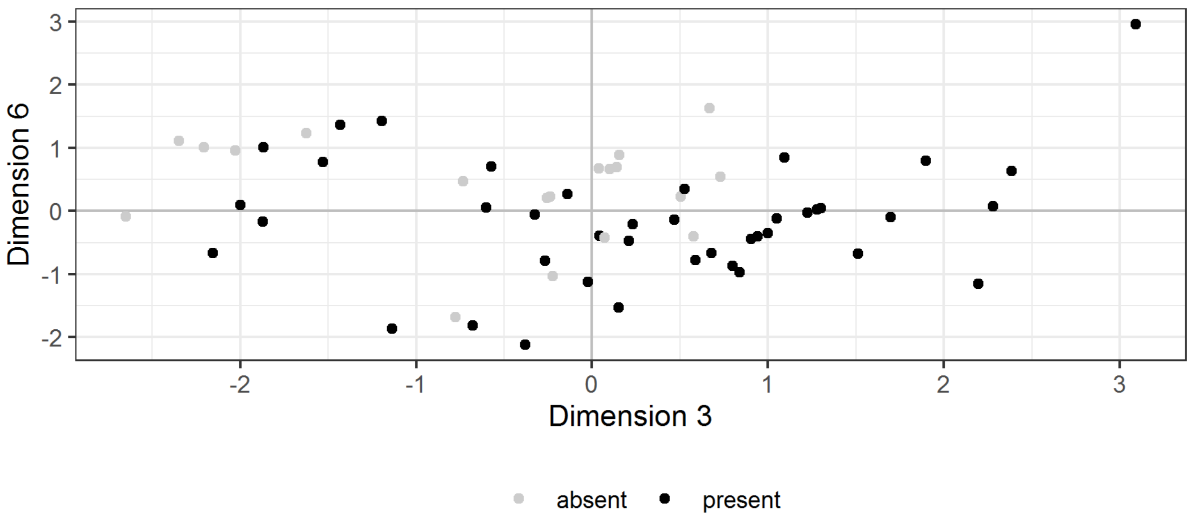

Pathogenes association

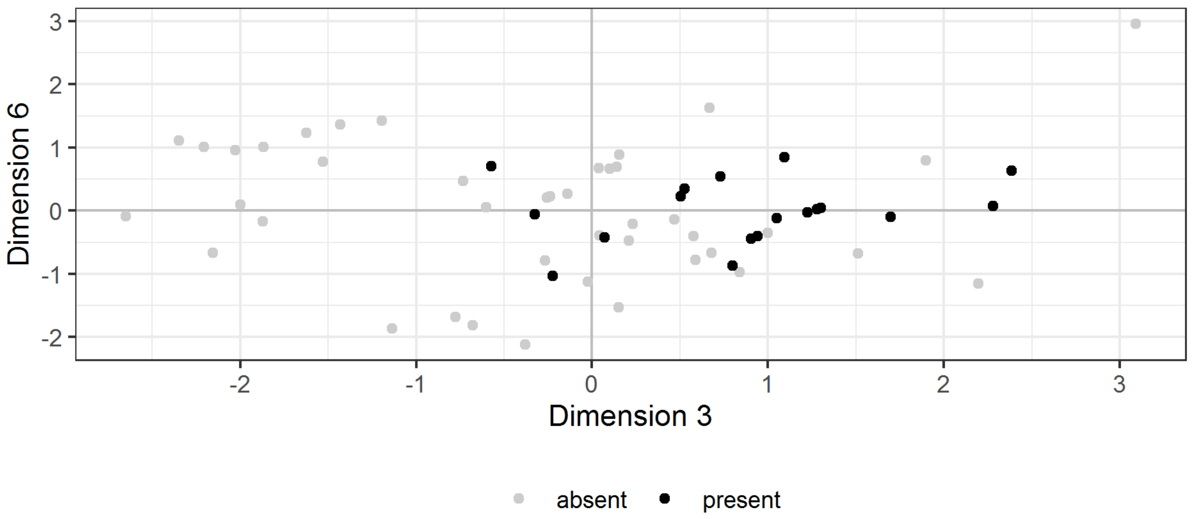

Phleophagy

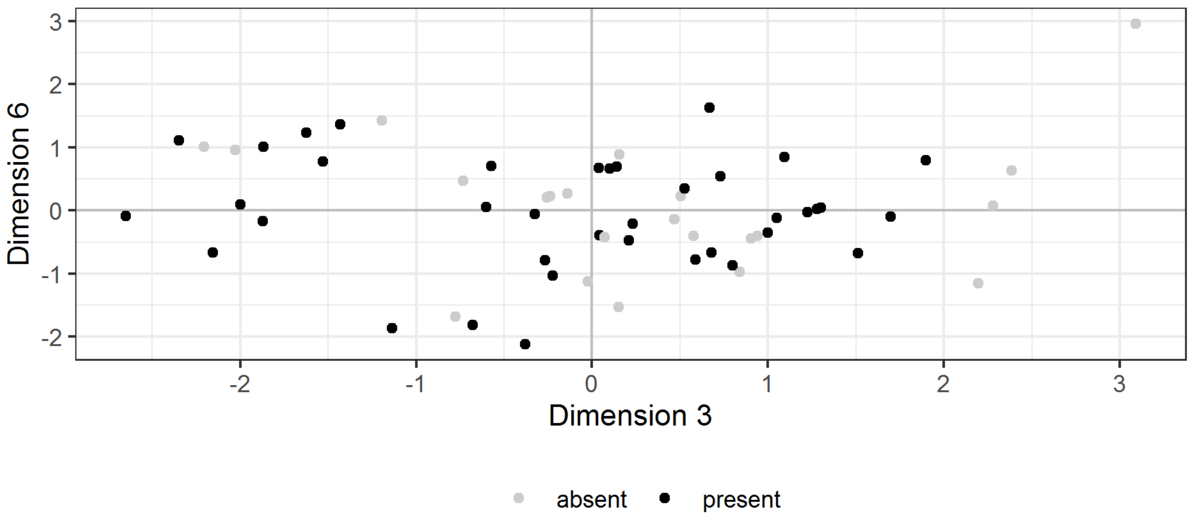

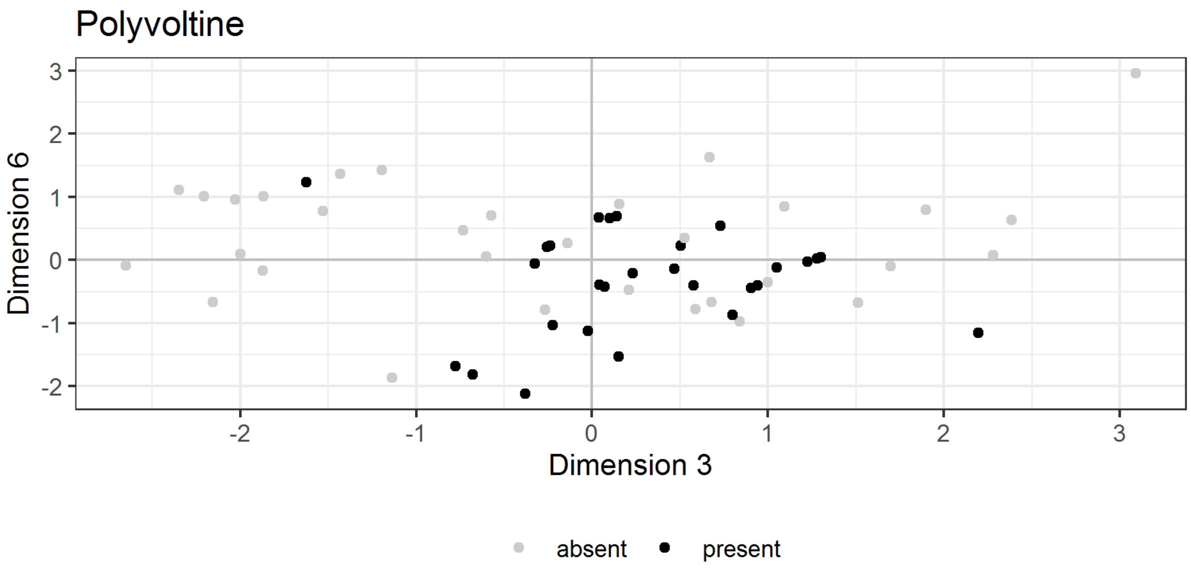

Polyvoltine
3
2
1
0
-1
-2
Dimension 6
-2
-1
0
1
2
3
Dimension 3
absent
present

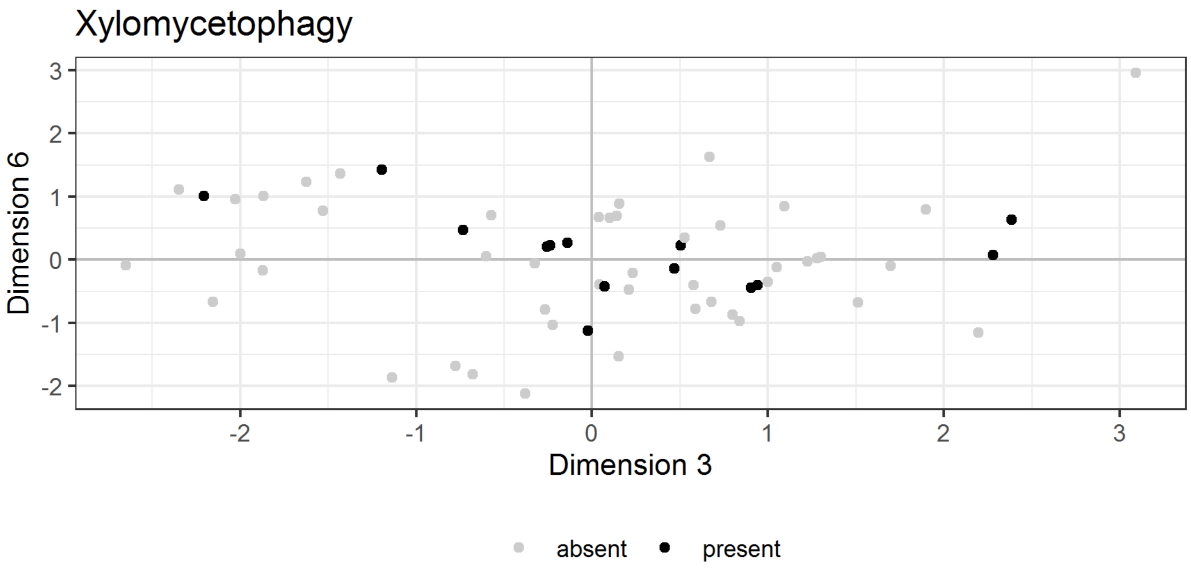

Xylomycetophagy
3
2
1
0
-1
-2
Dimension 6
-2
-1
0
1
2
3
Dimension 3
absent
present

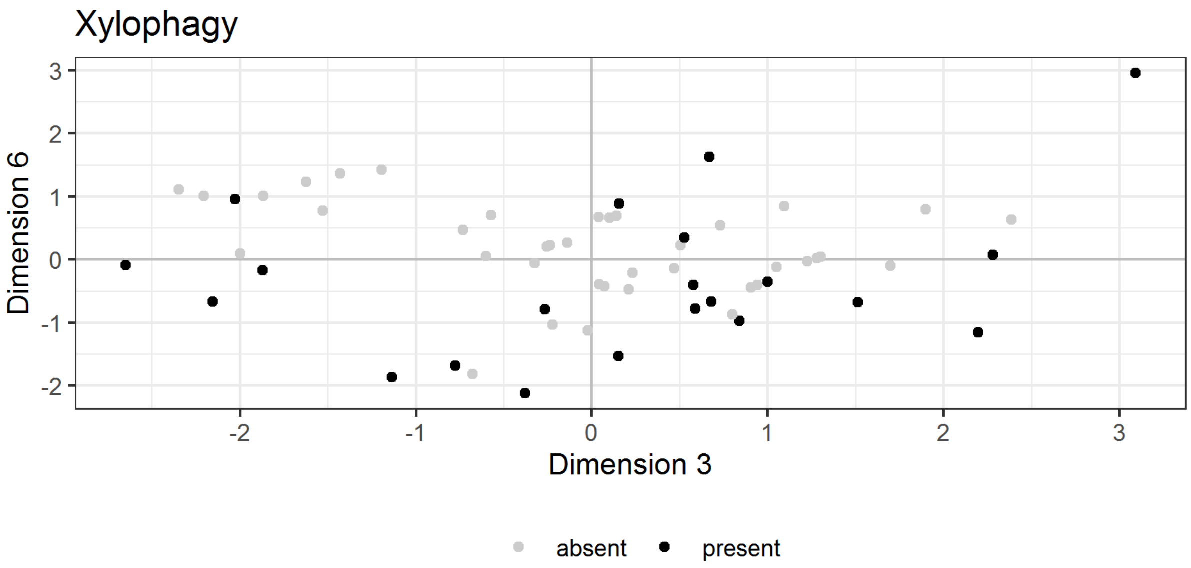

Xylophagy
3
2
1
0
-1
-2
Dimension 6
-2
-1
0
1
2
3
Dimension 3
absent
present
