# Supplementary material for: Biological Strategies of Invasive Bark Beetles and Borers Species
Source: Insects. 2021 Apr 20;12(4):367. doi: 10.3390/insects12040367 (PMC8074309; doi:10.3390/insects12040367)
Supplement: Supplementary file 1 [file insects-12-00367-s001.zip › Tables S2-S4.pdf]

Table S2

Results of fuzzy clustering and its comparison with PAM clusters. The numbers are probabilities of membership for given cluster. Probabilities < 0.2 are omitted

SPECIES	PAM CLUSTER	FUZZY CLUSTERS			
		1	2	3	4
A. monachus	*polyphagous*	0.47	0.27		
D. minutus	*intermediate*	0.22	0.39	0.21	
L. brunneus	*polyphagous*	0.51	0.23		
A. planipennis	*intermediate*	0.34	0.40	0.21	
L. festiva	*polyphagous*	0.66			
P. archon	*polyphagous*	0.43	0.30		
A. chinensis	*polyphagous*	0.74			
A. glabripennis	*polyphagous*	0.76			
A. bungii	*polyphagous*	0.34	0.29	0.27	
C. rufipenne	*polyphagous*	0.52	0.25		
P. recurva	*intermediate*		0.68		
P. semipunctata	*intermediate*		0.69		
P. hilaris	*polyphagous*	0.45	0.34		
R. rusticus	*polyphagous*	0.75			
T. fuscum	*aggressive*		0.27	0.46	
T. campestris	*polyphagous*	0.72			
X. chinensis	*polyphagous*	0.31	0.36	0.23	
A. dispar	*inbred*				0.62
C. lapathi	*polyphagous*	0.78			
D. micans	*inbred*	0.22	0.28		0.33
D. valens	*aggressive*		0.21	0.62	
E. destruens	*inbred*				0.93
H. ater	*intermediate*		0.39	0.36	
H. opacus	*intermediate*		0.55	0.25	
H. ligniperda	*intermediate*		0.69		
I. cembrae	*aggressive*			0.83	
M. mutatus	*polyphagous*	0.35	0.24	0.20	0.21
O. erosus	*aggressive*		0.35	0.49	
P. armatus	*aggressive*		0.37	0.40	
P. rudis	*intermediate*		0.63	0.20	
P. juglandis	*aggressive*			0.69	
P. proximus	*aggressive*			0.84	
R. ferrugineus	*intermediate*	0.21	0.35	0.29	
S. multistriatus	*aggressive*			0.85	
S. schevyrewi	*aggressive*		0.35	0.48	
T. piniperda	*aggressive*		0.21	0.64	
X. saxesenii	*inbred*				0.76
X. affinis	*inbred*				0.78
X. glabratus	*inbred*				0.79
X. perforans	*inbred*				0.93
X. similis	*inbred*				0.89
X. compactus	*inbred*				0.89
X. crassiusculus	*inbred*				0.93
X. germanus	*inbred*				0.78
X. morigerus	*inbred*				0.92
S. noctilio	*aggressive*	0.28	0.25	0.29	
T. fuscicornis	*inbred*	0.3	0.26		0.26

Table S3

Contributions of traits to principal components for FAMD (invasive species only)

TRAITS	DIM.1	DIM.2	DIM.3	DIM.4	DIM.5
**Fecundity**	5.7	0.9	13.6	0.6	27.5
**Phleophagy**	10.4	6.5	3.0	6.2	1.5
**Xylophagy**	5.6	11.5	9.3	0.0	0.6
**Xylomycetophagy**	15.0	3.3	0.2	2.7	6.4
**Inbreeding**	17.8	1.5	0.1	0.0	0.3
**Symbiotic plant pathogenes**	1.1	6.3	14.2	19.9	5.1
**Long-range sex pheromones**	2.6	9.2	9.0	2.9	2.8
**Aggregation pheromones**	1.6	15.0	0.0	9.4	20.0
**Maturation feeding**	3.2	4.8	9.0	15.5	2.6
**Food spectra**	3.5	21.4	17.1	38.6	20.0
**Flyless**	17.8	1.2	0.6	0.2	0.8
**Polyvoltinism**	5.6	7.8	4.4	3.4	5.9
**Monovoltinism**	4.4	1.6	19.2	0.8	5.1
**Multy-year life cycle**	5.7	9.1	0.5	0.0	1.2

Table S4

Contributions of traits to principal components for FAMD (invasive vs. non-invasive species)

TRAITS	DIM.1	DIM.2	DIM.3	DIM.4	DIM.5	DIM.6
**Fecundity**	3.4	3.1	13.3	0.5	9.8	13.1
**Phleophagy**	8.7	10.4	1.9	3.1	6.1	0.4
**Xylophagy**	6.6	10.3	0.0	0.2	7.3	9.4
**Xylomycetophagy**	15.6	3.5	0.4	2.5	0.3	1.3
**Inbreeding**	18.2	2.1	0.1	0.2	0.9	0.0
**Symbiotic plant pathogenes**	1.2	6.9	11.9	21.1	3.8	0.0
**Long-range sex pheromones**	2.7	8.5	14.3	2.9	0.1	1.1
**Aggregation pheromones**	0.7	13.5	7.5	11.3	2.0	5.8
**Maturation feeding**	3.7	4.4	15.3	5.2	0.6	0.5
**Food spectra**	3.0	19.1	3.6	41.9	10.0	19.1
**Flyless**	17.2	2.5	0.1	0.1	0.8	1.4
**Polyvoltinism**	6.8	6.3	2.6	3.4	10.6	11.8
**Monovoltinism**	3.2	0.0	4.3	0.1	45.7	8.9
**Multy-year life cycle**	6.8	8.4	1.5	0.2	0.1	0.7
**Invasiveness**	2.3	0.9	23.2	7.5	1.8	26.3
